# Supplementary material for: Identification of proximal SUMO-dependent interactors using SUMO-ID
Source: Nat Commun. 2021 Nov 18;12:6671. doi: 10.1038/s41467-021-26807-6 (PMC8602451; doi:10.1038/s41467-021-26807-6)
Supplement: Supplementary file 1 — Supplementary Information [file 41467_2021_26807_MOESM1_ESM.pdf]

Identification of proximal SUMO-dependent interactors using SUMO-ID

Barroso-Gomila et al.

SUPPLEMENTARY INFORMATION

Supplementary Note 1:

The biotin binding pocket of BirA is composed of three β-strands (strands 5, 8 and 9), the N-terminus of helix E and the 110-128 loop (Fig. 2a). Particularly, amino acids within the 110-128 loop have crucial interactions with biotin[1, 2]. The carbonyl oxygen of biotin hydrogen bonds the amide nitrogen of Arg-116. The two ureido nitrogens found in biotin form hydrogen bonds with carbonyl oxygens in Gln-112 and Arg-116. The carboxyl group of the valeric acid bonds to the backbone amide of Arg-118. Trp-123 also participates in the binding through hydrophobic interactions with the hydrocarbon tail of the valeric acid. It is presumable that disruption of such essential loop would completely inactivate the enzyme, but it would also affect the reconstitution efficiency.

Biotin extends along the β-sheet formed by strands 5, 8 and 9 (Fig. 2a). The thiophene ring contacts glycine-186, 204 and 205 residues and Leu-188 interacts with the hydrocarbon tail through hydrophobic interactions. Lys-183 participates in biotin binding by forming a hydrogen bond with the carboxyl group of valeric acid. Interactions between amino acids forming the β-sheet and the 110-128 loop are also found, and they might participate to stabilize the biotin binding pocket. It is noteworthy that upon biotin binding, Glu-190 forms a salt bridge with Arg-116[1]. We observed that the 193-199 loop connects the β-strands 8 and 9. Residues within this loop are partially or completely disordered in the apoBirA structure, while they become visible upon biotin binding[2]. Thus, splitting TurboID at this loop might abrogate the biotin binding of the resulting TurboID fragments, while the central 110-128 loop would not be affected. We decided to split TurboID at T194/G195.

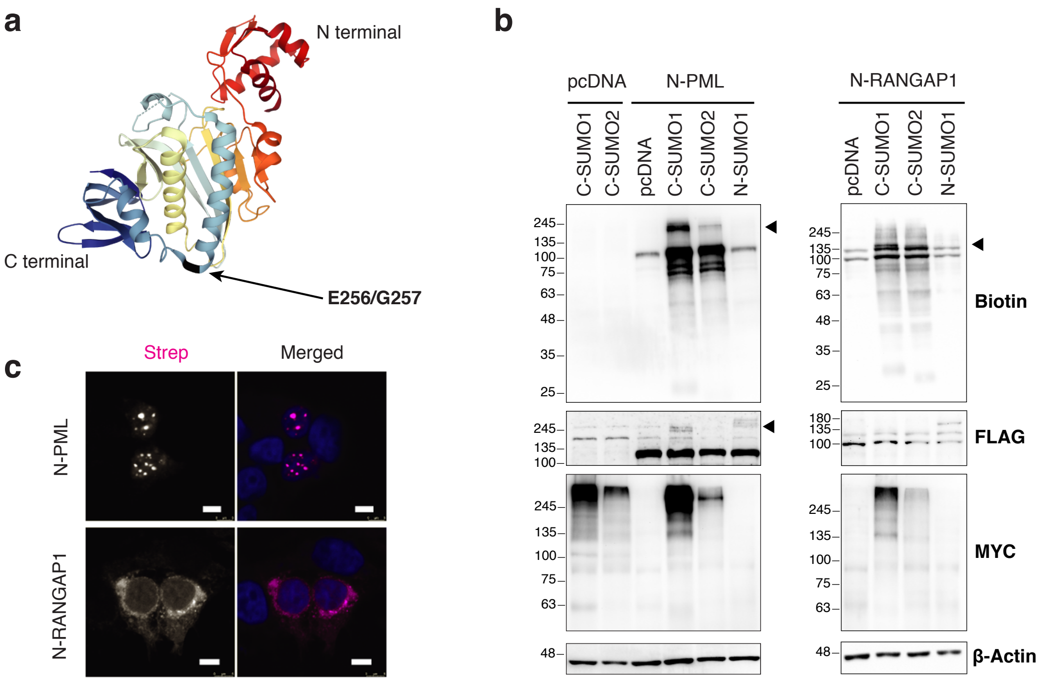

<u>Supplementary Fig. 1:</u> E256/G257 Split-TurboID is not suitable for SUMO-ID studies. (**a**) Structure of the *E. coli* BirA (PDB ID: 1HXD[2, 3] [http://doi.org/10.2210/pdb1HXD/pdb]) depicting the E256/G257 split point. (**b**) Western blot of HEK293FT cells that were transiently transfected with combinations of the FLAG-NTurboID^{256} (N) or MYC-CTurboID^{257} (C) fused to PML, RANGAP1 or SUMO1/2 and treated with 50 µM of biotin for 16 hours. Black arrowheads indicate SUMO-ID activity derived from MYC-CTurboID^{257}-SUMOylated FLAG-NTurboID^{256}-substrates. A high background biotinylating activity was observed when transfecting NTurboID^{256} alone. Data are representative of 2 independent transfection experiments with similar results. Molecular weight markers are shown to the left of the blots in kDa. Source data are provided in the Source Data file. (**c**) Immunostainings of transiently transfected HEK293FT cells treated with 50 µM of biotin for 16 hours, showing the background biotinylating activity of the FLAG-NTurboID^{256} alone. Nuclei are stained with DAPI (blue) and biotinylated material with fluorescent streptavidin (Strep, magenta). Black and white panel shows the magenta channel alone. Images are representative of 3 independent transfection experiments performed on cover slips. Scale bar: 5 µm.

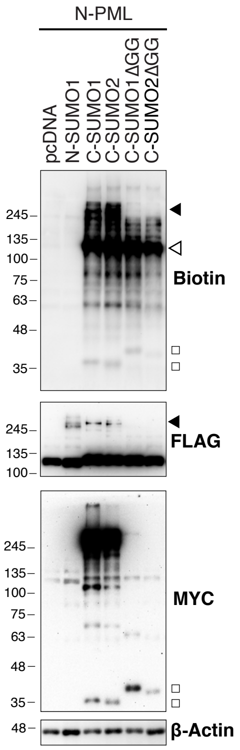

<u>Supplementary Fig. 2:</u> Free biotinylated C-SUMOs appear at long labelling times. Western blot of HEK293FT co-transfected with FLAG-N-PML and different combinations of FLAG-N- or MYC-C-SUMO^{WT} or -SUMO$^{\Delta GG}$. Cells were treated with 50 µM of biotin for 16 hours. White squares indicate free/unconjugated C-SUMOs. White arrowheads point to SUMO-SIM interaction mediated SUMO-ID. Black arrowheads show PML-SUMOylation derived SUMO-ID. Data are representative of 4 independent transfection experiments with similar results. Molecular weight markers are shown to the left of the blots in kDa. Source data are provided in the Source Data file.

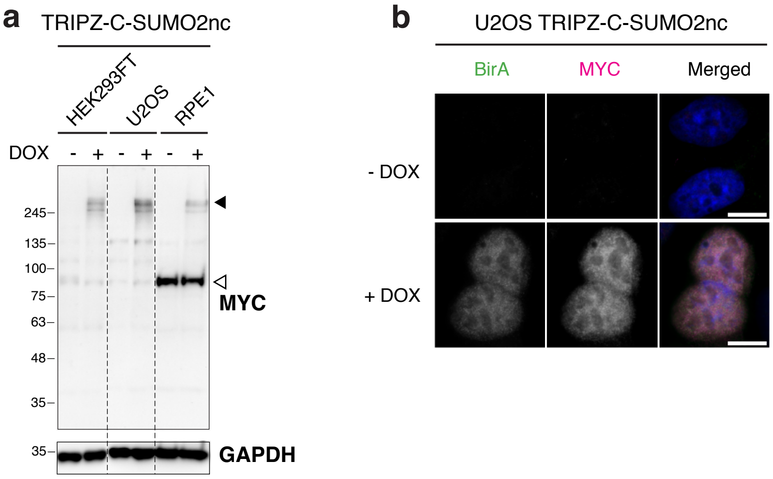

<u>**Supplementary Fig. 3:**</u> **Generation and validation of doxycycline inducible TRIPZ-MYC-C-SUMO2nc stable cell lines.** (**a**) Western blot showing the doxycycline induction dependency and the correct incorporation of MYC-C-SUMO2nc (black arrowhead) in 3 different stable cell lines. Doxycycline induction was determined to 1 µg/mL for 24 hours. The white arrowhead indicates unspecific signal detected in RPE1 cells. Dotted lines indicate a cut in the same blot. Results are representative of the correct induction that was regularly checked. Molecular weight markers are shown to the left of the blots in kDa. Source data are provided in the Source Data file. (**b**) Confocal microscopy showing the correct nuclear staining of MYC-C-SUMO2nc after 1 µg/mL of doxycycline treatment for 24 hours. Nuclei are stained with DAPI (blue), MYC in magenta. BirA antibody recognizes C (green). Black and white panels show the green and magenta channels alone. Images are representative of 3 independent doxycycline inductions. Scale bar 10 µm.

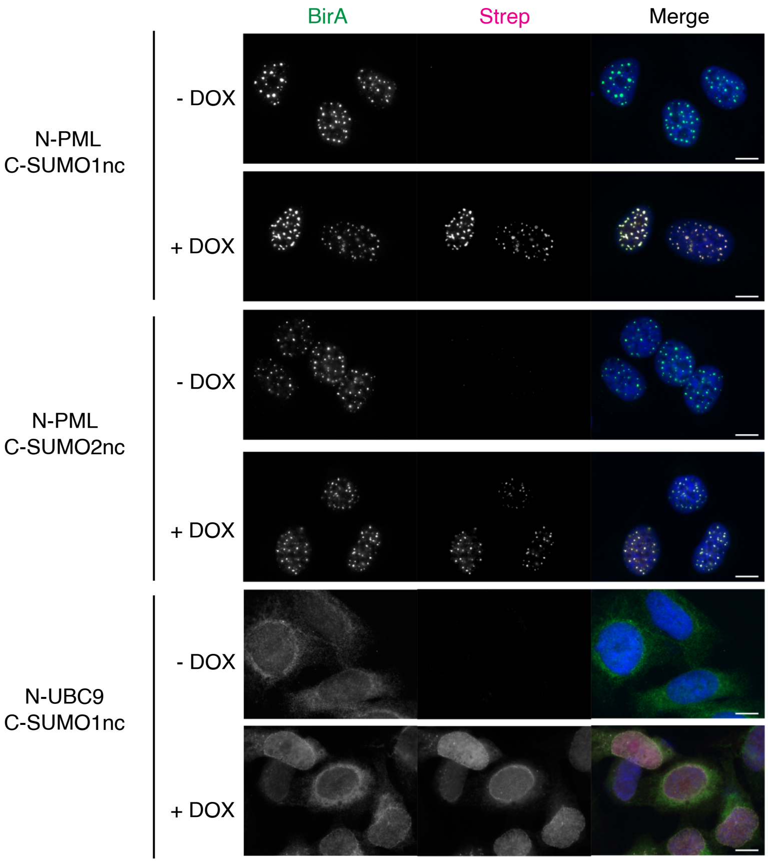

<u>Supplementary Fig. 4:</u> Localization of biotinylated interactors by SUMO-ID. Immunofluorescence of U2OS FLAG-N-PML / TRIPZ-MYC-C-SUMO1/2nc double stable cell lines after 2 hours of biotin treatment (50 µM). Cells were treated or not with 1 µg/mL of doxycycline for 24 hours to induce MYC-C-SUMO1/2nc expression. Colocalization of the biotinylated substrates and FLAG-N-PML signal within PML NBs was confirmed. U2OS FLAG-N-UBC9/ MYC-C-SUMO1nc double stable cell line was used as a specificity control. Nuclei are stained with DAPI (blue) and biotinylated material with fluorescent streptavidin (Strep, magenta). BirA antibody recognizes both N and C (green). Black and white panels show the single green and magenta channels. Images are representative of 3 independent experiments performed on cover slips. Scale bar: 10 µm.

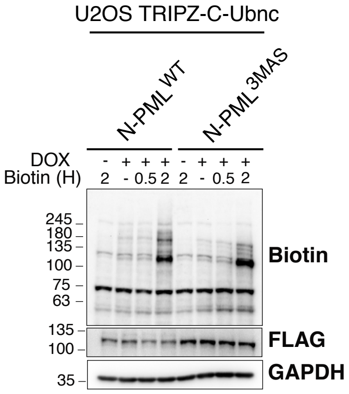

<u>Supplementary Fig. 5:</u> Ub-ID biotinylation activity is positive for both PML^{WT} and PML^{3MAS}. Western blot of U2OS TRIPZ-MYC-C-Ubnc / FLAG-N-PML^{WT} or the SUMO/SIM mutant PML^{3MAS}. Doxycycline was added or not at 1 µg/mL for 24 hours. 50 µM of biotin was added at the indicated time-points. In contrast to SUMO-ID, PML Ub-ID showed positive biotinylation activity in both WT and SUMO/SIM mutant forms of PML. Data are representative of 2 independent experiments performed on the same double stable cell lines. Molecular weight markers are shown to the left of the blots in kDa. Source data are provided in the Source Data file.

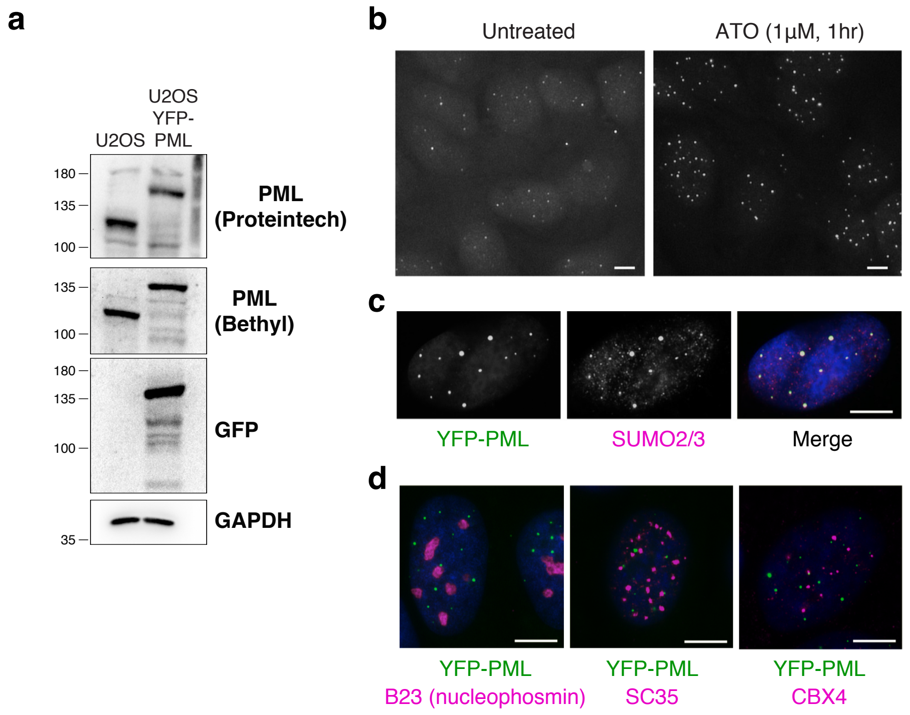

<u>**Supplementary Fig. 6:**</u> **Characterization of U2OS YFP-PML knock-in cell line.** Single cell knock-in clones were analyzed by PCR, sequencing, and western blotting. Used in this study is clone 3.3, which has a homozygous insertion of YFP at the 5' end of PML. (**a**) Western blot analysis of the parental U2OS cells and YFP-PML clone 3.3. Two different PML antibodies show that the major PML isoform migrates more slowly in YFP-PML cells. Specific labelling of the major, and more weakly expressed isoforms, is seen using GFP antibody. Results are representative of 3 independent biological replicates. Molecular weight markers are shown to the left of the blots in kDa. Source data are provided in the Source Data file. (**b**) YFP-PML concentrates in NBs upon ATO treatment (1 µM, 1 hr). (**c**) YFP-PML colocalizes with SUMO2/3 upon ATO treatment. (**d**) YFP-PML NBs do not colocalize with other nuclear condensates (B23, nucleoli; SC35, nuclear speckles; CBX4, Polycomb bodies). Nuclei are stained with DAPI (blue), YFP-PML is shown in green and the indicated proteins in magenta. Black and white panels in (c) show the single green and magenta channels. Images are representative of 3 independent biological replicates. Scale bar: 5 µm.

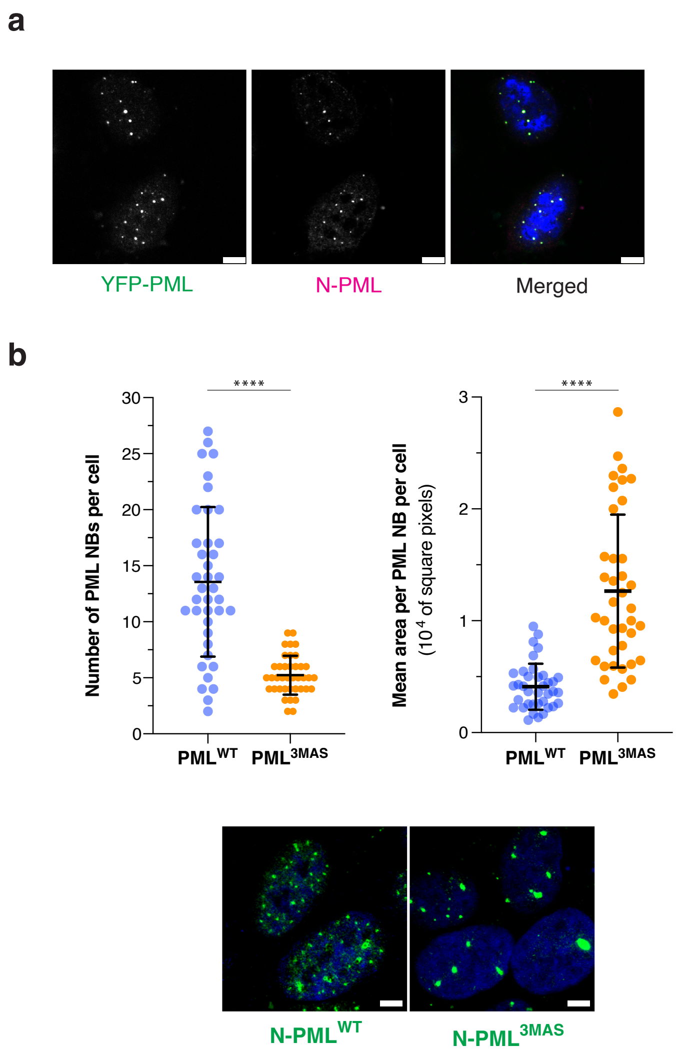

<u>Supplementary Fig. 7:</u> N-PML forms true PML NBs. (**a**) Confocal microscopy of U2OS YFP-PML cell line transfected with N-PML^{WT}. Nuclei are stained with DAPI (blue), YFP-PML in green and BirA antibody that recognizes N-PML in magenta. Colocalization of YFP-PML and N-PML at true PML-NBs is observed. Images are representative of 3 independent transfection experiments. Scale bar: 5 µm. (**b**) Quantification of the mean area and the number of PML NBs per cell of n = 40 U2OS cells stably expressing FLAG-N-PML^{WT} or -PML^{3MAS} over 4 independent replicates. Data are presented as mean values +/- SD. N-PML^{WT} NBs appeared to be smaller and more abundant than NB-like bodies formed by N-PML^{3MAS}. Statistical analyses were performed by two-sided unpaired *t*-test with Welch's correction: **** $p < 0.0001$. Nuclei are stained with DAPI (blue) and BirA antibody recognizes N (green). Source data are provided in the Source Data file. Scale bar: 5 µm.

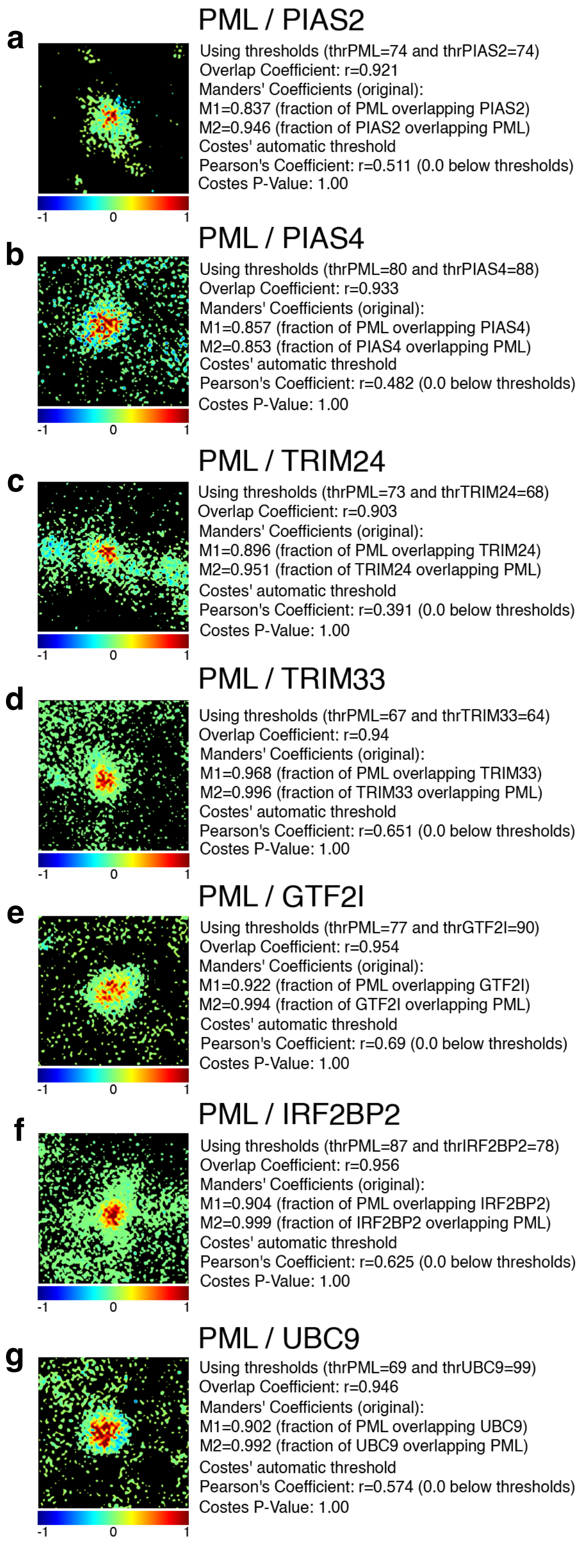

Supplementary Fig. 8: Colocalization of selected interactors with PML. (a-g). Colocalization image analyses of the selected regions marked with dotted-line squares in the Fig. 5 images. Color code below each image corresponds to normalized mean deviation product (nMDP), where values above 0 indicate colocalization. Parameters indicated to the right were calculated using autothreshold or the Costes' automatic threshold options. Costes *p*-value: 1.00 indicates colocalization.

Supplementary Table 1: **Details of used constructs.**

Plasmid Name	Resistance	Plasmid Backbone	Plasmid Insert	Plasmid Insert	Sources/cloning notes
Lenti-EFS-NTurboID[255]-GSQ-RANGAP1-P2A-BLAST	AMP	Lenti-EFS-P2A-blast	FLAG-NTurboID[255] (BshT1-Asc1)	RANGAP1 (EcoR1-Not1)	Based on Lenti-Cas9-blast (Addgene 52962; F. Zhang); Cas9 removed BshT1-BamH1; Elements: EFS-BshT1-Asc1-GSQ-EcoR1-Not1-BamH1-P2A-blast; Sources: pEGFP-C2 RanGAP (Addgene #13378; M. Dasso); V5-TurboID-NES_pCDNA3 (Addgene #107169; A. Ting)
Lenti-EFS- NTurboID[255]-GSQ-PMLIVa-P2A-BLAST*	AMP	Lenti-EFS-P2A-blast	FLAG-NTurboID[255]	PMLiva	Based on Lenti-Cas9-blast; Source: pCMV Tag 2B Flag PML4 WT (PMID 17081985); V5-TurboID-NES_pCDNA3
Lenti-EFS-CTurboID[256]-GSQ-SUMO1-T2A-PURO	AMP	Lenti-EFS-T2A-puro	MYC-CTurboID[256]	SUMO1	Based on Lenti-T2A-puro (built from Lenti-Cas9-blast); Cas9 removed BshT1-BamH1; inserted T2A-puro; Elements: EFS-BshT1-Asc1-GSQ-EcoR1-Not1-BamH1-T2A-puro; SUMO1 source: hTERT-RPE1 cDNA;
Lenti-EFS-CTurboID[256]-GSQ-SUMO2-T2A-PURO	AMP	Lenti-EFS-T2A-puro	MYC-CTurboID[256]	SUMO2	Based on Lenti-T2A-puro; Elements: EFS-BshT1-Asc1-GSQ-EcoR1-Not1-BamH1-T2A-puro; SUMO2 source: hTERT-RPE1 cDNA; V5-TurboID-NES_pCDNA3 (Addgene #107169; A. Ting)
Lenti-EFS- NTurboID[255]-GSQ-SUMO1-P2A-BLAST	AMP	Lenti-EFS-P2A-blast	FLAG-NTurboID[255]	SUMO1	Based on Lenti-Cas9-blast; SUMO1 source: hTERT-RPE1 cDNA; V5-TurboID-NES_pCDNA3
Lenti-EFS- NTurboID[194]-GSQ-RANGAP1-P2A-BLAST*	AMP	Lenti-EFS-P2A-blast	FLAG-NTurboID[194]	RANGAP1	Based on Lenti-Cas9-blast; RANGAP1 source pEGFP-C2 RanGAP (Addgene #13378; M. Dasso); V5-TurboID-NES_pCDNA3
Lenti-EFS- NTurboID[194]-GSQ-PMLivaWT-P2A-BLAST*	AMP	Lenti-EFS-P2A-blast	FLAG-NTurboID[194]	PMLivaWT	Based on Lenti-Cas9-blast; PMLiva source: pCMV Tag 2B Flag PML4 WT (PMID 17081985); V5-TurboID-NES_pCDNA3
Lenti-EFS- NTurboID[194]-GSQ-PMLiva3MAS-P2A-BLAST*	AMP	Lenti-EFS-P2A-blast	FLAG-NTurboID[194]	PMLiva3MAS	Based on Lenti-Cas9-blast; PMLiva source: pCMV Tag 2B Flag PML4 3MAS (PMID 17081985); mutation of 3 SUMOylation sites and SIM motif; V5-TurboID-NES_pCDNA3
Lenti-EFS- NTurboID[194]-GSQ-UBC9-P2A-BLAST	AMP	Lenti-EFS-P2A-blast	FLAG-NTurboID[194]	UBC9	Based on Lenti-Cas9-blast; UBC9 source: CAG-bioSUMO2-2A-BirA-2A-UBC9 (PMID 28098257); V5-TurboID-NES_pCDNA3

Lenti-EFS-CTurboID[195]-GSQ-SUMO1-T2A-PURO	AMP	Lenti-EFS-T2A-puro	MYC-CTurboID[195]	SUMO1	Based on Lenti-T2A-puro; Elements: EFS-BshT1-Asc1-GSQ-EcoR1-Not1-BamH1-T2A-puro; SUMO1 source: hTERT-RPE1 cDNA; V5-TurboID-NES_pCDNA3
Lenti-EFS-CTurboID[195]-GSQ-SUMO2-T2A-PURO	AMP	Lenti-EFS-T2A-puro	MYC-CTurboID[195]	SUMO2	Based on Lenti-T2A-puro; Elements: EFS-BshT1-Asc1-GSQ-EcoR1-Not1-BamH1-T2A-puro; SUMO2 source: hTERT-RPE1 cDNA; V5-TurboID-NES_pCDNA3
Lenti-EFS-NTurboID[194]-GSQ-SUMO1-P2A-BLAST	AMP	Lenti-EFS-P2A-blast	FLAG-NTurboID[194]	SUMO1	Based on Lenti-Cas9-blast; SUMO1 source: hTERT-RPE1 cDNA; V5-TurboID-NES_pCDNA3
Lenti-EFS-NTurboID[194]-GSQ-FRB-P2A-BLAST*	AMP	Lenti-EFS-P2A-blast	FLAG-NTurboID[194]	FRB	Based on Lenti-Cas9-blast; FRB source pSF3-Flag-CBir-FRB_Myc-NBir-FKBP (Addgene# 90003; J. Bethune); V5-TurboID-NES_pCDNA3
Lenti-EFS- CTurboID[195]-GSQ-FKBP-T2A-PURO*	AMP	Lenti-EFS-T2A-puro	MYC-CTurboID[195]	FKBP	Based on Lenti-T2A-puro; Elements: EFS-BshT1-Asc1-GSQ-EcoR1-Not1-BamH1-T2A-puro; FKBP source pSF3-Flag-CBir-FRB_Myc-NBir-FKBP (Addgene# 90003; J. Bethune); V5-TurboID-NES_pCDNA3
Lenti-EFS- CTurboID[195]-GSQ-SUMO1DGG-T2A-PURO	AMP	Lenti-EFS-T2A-puro	MYC-CTurboID[195]	SUMO1DGG	Based on Lenti-T2A-puro; Elements: EFS-BshT1-Asc1-GSQ-EcoR1-Not1-BamH1-T2A-puro; SUMO1 source: Lenti-EFS- CTurboID[256]-GSQ-SUMO1-T2A-PURO; non-conjugatable
Lenti-EFS- CTurboID[195]-GSQ-SUMO2DGG-T2A-PURO	AMP	Lenti-EFS-T2A-puro	MYC-CTurboID[195]	SUMO2DGG	Based on Lenti-T2A-puro; Elements: EFS-BshT1-Asc1-GSQ-EcoR1-Not1-BamH1-T2A-puro; SUMO2 source: Lenti-EFS- CTurboID[256]-GSQ-SUMO2-T2A-PURO; non-conjugatable
CMV-bioSUMO1wt	KAN	EYFP-N1	bioSUMO1wt		Replaced EYFP (EcoR1-Not1) with Avi-tagged SUMO1; SUMO1 source: Lenti-EFS-CTurboID[256]-GSQ-SUMO1-T2A-PURO; EYFP-N1 (Clontech/Takara)
CMV-bioSUMO2wt	KAN	EYFP-N1	bioSUMO2wt		Replaced EYFP (EcoR1-Not1) with Avi-tagged SUMO2; SUMO2 source: Lenti-EFS-CTurboID[256]-GSQ-SUMO2-T2A-PURO
CMV-bioUBwt	KAN	EYFP-N1	bioUbwt		Replaced EYFP (EcoR1-Not1) with Avi-tagged Ubiquitin UB; UB source: CAG-bioUB (PMID 28098257)

CMV-bioSUMO1nc	KAN	EYFP-N1	bioSUMO1nc		Replaced EYFP (EcoR1-Not1) with Avi-tagged SUMO1nc; incorporates mutation Q94P to suppress deSUMOylation from substrates and terminates in Gly-Gly to bypass initial protease activation step
CMV-bioSUMO2nc	KAN	EYFP-N1	bioSUMO2nc		Replaced EYFP (EcoR1-Not1) with Avi-tagged SUMO2nc; incorporates mutation Q90P to suppress deSUMOylation from substrates and terminates in Gly-Gly to bypass initial protease activation step
CMV-bioUBnc	KAN	EYFP-N1	bioUbnc		Replaced EYFP (EcoR1-Not1) with Avi-tagged Ubiquitin UBnc; incorporates mutation L73P to suppress deubiquitylation from substrates and terminates in Gly-Gly to bypass initial protease activation step
TRIPZ- CTurboID^{195}-GSQ-SUMO1nc-PURO*	AMP	TRIPZ	MYC-CTurboID^{195}	SUMO1nc	Based on TRIPZ (lentiviral all-in-one doxycyline-inducible vector; OpenBiosystems/Thermo); Gibson-cloned elements into BshT1-Mlu1 digested vector (removing TurboRFP and shRNA); Source: CMV-bioSUMO1nc; V5-TurboID-NES_pCDNA3
TRIPZ- CTurboID^{195}-GSQ-SUMO2nc-PURO*	AMP	TRIPZ	MYC-CTurboID^{195}	SUMO2nc	Based on TRIPZ (lentiviral all-in-one doxycyline-inducible vector; OpenBiosystems/Thermo); Gibson-cloned elements into BshT1-Mlu1 digested vector (removing TurboRFP and shRNA); Source: CMV-bioSUMO2nc; V5-TurboID-NES_pCDNA3
TRIPZ- CTurboID^{195}-GSQ-Ubnc-PURO*	AMP	TRIPZ	MYC-CTurboID^{195}	Ubnc	Based on TRIPZ (lentiviral all-in-one doxycyline-inducible vector; OpenBiosystems/Thermo); Gibson-cloned elements into BshT1-Mlu1 digested vector (removing TurboRFP and shRNA); Source: CMV-bioUBnc; V5-TurboID-NES_pCDNA3
Lenti-EFS-FLTurboID-GSQ-PMLIVa^{WT}-P2A-BLAST*	AMP	Lenti-EFS-P2A-blast	FLAG-FLTurboID	PMLIVa^{WT}	Based on Lenti-Cas9-blast; PMLIVa source: pCMV Tag 2B Flag PML4 WT (PMID 17081985); FLTurboID=Full-length TurboID; V5-TurboID-NES_pCDNA3
Lenti-EFS-FLTurboID-GSQ-PMLIVa^{3MAS}-P2A-BLAST*	AMP	Lenti-EFS-P2A-blast	FLAG-FLTurboID	PMLIVa^{3MAS}	Based on Lenti-Cas9-blast; PMLIVa source: pCMV Tag 2B Flag PML4 3MAS (PMID 17081985); mutation of 3 SUMOylation sites and SIM motif; FLTurboID=Full-length TurboID; V5-TurboID-NES_pCDNA3
Lenti-EFS-FLTurboID-GSQ-RBXN-P2A-BLAST*	AMP	Lenti-EFS-P2A-blast	FLAG-FLTurboID	RBXN-linker	Based on Lenti-Cas9-blast; FLTurboID=Full-length TurboID; V5-TurboID-NES_pCDNA3; used as TurboID-only vector; RBXN is EcoR1, BsiW1, Xba1, Not1 multi-cloning site linker; encodes EFRTSRGGR
pcDNA3 (commercial)	AMP	pcDNA3	empty		Invitrogen; transfection control

Lenti-EFS-NTurboID^{194}-GSQ-SALL1^{wt}-P2A-BLAST*	AMP	Lenti-EFS-P2A-blast	FLAG-NTurboID^{194}	SALL1^{wt}	Based on Lenti-Cas9-blast; SALL1 source CMV-BioID-SALL1(full-length) (PMID 29395072); V5-TurboID-NES_pCDNA3
Lenti-EFS- NTurboID^{194}-GSQ-SALL1$^{\Delta SUMO}$-P2A-BLAST*	AMP	Lenti-EFS-P2A-blast	FLAG-NTurboID^{194}	SALL1$^{\Delta SUMO}$	Based on Lenti-Cas9-blast; SALL1 source CMV-BioID-SALL1(full-length) (PMID 29395072); V5-TurboID-NES_pCDNA3; 4 predicted SUMOylation sites mutated (K571R, K582R, K982R, K1086R).
Lenti-EFS- NTurboID^{194}-GSQ-TP53-P2A-BLAST*	AMP	Lenti-EFS-P2A-blast	FLAG-NTurboID^{194}	TP53	Based on Lenti-Cas9-blast; TP53 source: hTERT-RPE1 cDNA; V5-TurboID-NES_pCDNA3

Supplementary Table 2: **Oligonucleotides sequences and uses.**

Oligo name	Sequence (5'-3')	Used to construct following plasmids or intermediates
TID.RANGAP1.for	GGCGGGCAAATTTCTTACGCAAGTAGAGGGGAATTCATGGCCTCGGAAGACATTGCC	Lenti-EFS-NTID^{255}-GSQ-RANGAP1-P2A-BLAST
TID.RANGAP1.rev	GAAGTTTGTTGCGCCGGATCCGCGGCCGCCGACCTTGTACAGCGTCTGCAGCAG	Lenti-EFS-NTID^{255}-GSQ-RANGAP1-P2A-BLAST
EFS.FLAG.TurboID_M1.for	CAACGGGTTTGCCGCCAGAACACAGGACCGGTGCCACCATGGACTACAAAGACGATGACGACAAGGGCTCTAAAGACAATACTGTGCCTCTGAAGCTG	Lenti-EFS-NTID^{255}-GSQ-RANGAP1-P2A-BLAST
TurboID_255.rev	AATTTGCCCGCCTCCGGAAGATCCCCCGCCGGCGCGCCCTTCCTGCTCGAACAGTTCCAGGGC	Lenti-EFS-NTID^{255}-GSQ-RANGAP1-P2A-BLAST
EFS_PMLiva.for	CAACGGGTTTGCCGCCAGAACACAGGACCGGTGCCACCATGGAGCCTGCACCCGCCCGATC	Lenti-EFS-NTID^{255}-GSQ-PMLiva-P2A-BLAST; Lenti-EFS-NTID^{255}-GSQ-PMLiva3MAS-P2A-BLAST
GSQ_PMLiva.rev	AATTTGCCCGCCTCCGGAAGATCCCCCGCCGGCGCGCCCAATTAGAAAGGGGTGGGGGTAGCCC	Lenti-EFS-NTID^{255}-GSQ-PMLiva-P2A-BLAST; Lenti-EFS-NTID^{255}-GSQ-PMLiva3MAS-P2A-BLAST
EFS.MYC.TurboID_256.for	CAACGGGTTTGCCGCCAGAACACAGGACCGGTGCCACCATGGAACAAAAACTCATCTCAGAAGAGGATCTGGGCTCTGGCCTGGCTCCATATCTGCCACGG	Lenti-EFS-CTID^{256}-GSQ-SUMO1-T2A-PURO
TurboID_K321.rev	AATTTGCCCGCCTCCGGAAGATCCCCCGCCGGCGCGCCCCTTTTCGGCAGACCGCAGACTGAT	Lenti-EFS-CTID^{256}-GSQ-SUMO1-T2A-PURO
CMV_BIO_for	ACTCAGATCTCGAGCTCAAGCTTCGAATTCGCCACCATGGGTTTGAATGACATA	CMV-bioSUMO1wt; CMV-bioSUMO2wt; CMV-bioSUMO1nc; CMV-bioSUMO2nc
N1_SUMO1_wt_rev	ATGTGGTATGGCTGATTATGATCTAGAGTCGCGGCCGCTTAACCCCCCGTTTGTTCCTGATA	CMV-bioSUMO1wt (used as template for Lenti-EFS/TRIPZ versions)
N1_SUMO1_nocut_rev	GGTATGGCTGATTATGATCTAGAGTCGCGGCCGCTTAACCACCCGTAGGTTCCTGATAAACTTCAATCACATCTTCTTC	CMV-bioSUMO1nc (used as template for Lenti-EFS/TRIPZ versions)
N1_SUMO2_wt_rev	ATGTGGTATGGCTGATTATGATCTAGAGTCGCGGCCGCTTAACCTCCCGTCTGCTGTTGGAA	CMV-bioSUMO2wt (used as template for Lenti-EFS/TRIPZ versions)
N1_SUMO2_nocut_rev	TGGTATGGCTGATTATGATCTAGAGTCGCGGCCGCTTAACCTCCCGTTGGCTGTTGGAACACATCAATTGTATCTTCAT	CMV-bioSUMO2nc (used as template for Lenti-EFS/TRIPZ versions)
GSG.R1.UBC9.for	CAAATTTCTTACGCAAGTAGAGGGGAATTCATGTCGGGGATCGCCCTC	Lenti-EFS-NTID^{194}-GSQ-UBC9-P2A-BLAST
UBC9.Not1.P2A.rev	GAAGTTTGTTGCGCCGGATCCGCGGCCGCCTGAGGGCGCAAACTTCTTGGC	Lenti-EFS-NTID^{194}-GSQ-UBC9-P2A-BLAST
GSQ_FRB.for	CAAATTTCTTACGCAAGTAGAGGGGAATTCATGATCCTCTGGCATGAGATGTGG	Lenti-EFS-NTID^{194}-GSQ-FRB-P2A-BLAST

FRB_P2A.rev	GAGAGAAGTTTGTTGCGCCGGATCCGCGGCCGCCTTTAATTAACTGCTTTGAGATTCGTCGGAACAC	Lenti-EFS-NTID[194]-GSQ-FRB-P2A-BLAST
GSQ_FKBP.for	CAAATTTCTTACGCAAGTAGAGGGGAATTCATGGGAGTGCAGGTGGAAACCATC	Lenti-EFS-CTID[195]-GSQ-FKBP-T2A-PURO
FKBP_T2A.rev	CTCTGCCCTCTCCGCTTCCGGATCCGCGGCCGCCACGCGTTTCCAGTTTTAGAAGCTCCAC	Lenti-EFS-CTID[195]-GSQ-FKBP-T2A-PURO
Not1.SUMO1dGG.rev	GATCGCGGCCGCTCGTTTGTTCCTGATAAACTTCAATCACATC	Lenti-EFS-CTID[195]-GSQ-SUMO1DGG-T2A-PURO
Not1.SUMO2dGG.v2.rev	GATCGCGGCCGCTCGTCTGCTGTTGGAACACATCAATTGTA	Lenti-EFS-CTID[195]-GSQ-SUMO2DGG-T2A-PURO
TID.SALL1.for	GAAGTTTGTTGCGCCGGATCCGCGGCCGCCACTCGTGACGATCTCCTTGCTGTCCTC	Lenti-EFS-NTID[194]-GSQ-SALL1wt-P2A-BLAST; Lenti-EFS-NTID[194]-GSQ-SALL1DSUMO-P2A-BLAST
TID.SALL1.rev	GGCGGGCAAATTTCTTACGCAAGTAGAGGGGAATTCATGGCCGAATTTACAAGCTACAAGGAGACG	Lenti-EFS-NTID[194]-GSQ-SALL1wt-P2A-BLAST; Lenti-EFS-NTID[194]-GSQ-SALL1DSUMO-P2A-BLAST
RBXN_linker.for	AATTCCGTACGTCTAGAGGC	Lenti-EFS-FLTID-GSQ-RBXN-P2A-BLAST (annealled and cloned into EcoR1-Not1 site)
RBXN_linker.rev	GGCCGCCTCTAGACGTACGG	Lenti-EFS-FLTID-GSQ-RBXN-P2A-BLAST (annealled and cloned into EcoR1-Not1 site)
GSQ.HsTP53.for	CAAATTTCTTACGCAAGTAGAGGGGAATTCATGGAGGAGCCGCAGTCAGATCCT	Lenti-EFS-NTID[194]-GSQ-TP53-P2A-BLAST
P2A.HsTP53.rev	GAGAGAAGTTTGTTGCGCCGGATCCGCGGCCGCCGTCTGAGTCAGGCCCTTCTGTCTT	Lenti-EFS-NTID[194]-GSQ-TP53-P2A-BLAST
TRIPZ.EFS.for	CAGAGCTCGTTTAGTGAACCGTCAGATCGCTTGCCGCCAGAACACAGGACCGGT	TRIPZ-CTID[195]-GSQ-SUMO1nc/SUMO2nc/UBnc-PURO (to shuttle from Lenti-EFS vectors into TRIPZ)
P2A.TRIPZ.rev	GCGCCAAAACCCGGCGCGGAGGCCACGCGTTCCGGCTTGTTTCAGCAGAGATCAGTTTGTTGCGCC	TRIPZ-CTID[195]-GSQ-SUMO1nc/SUMO2nc/UBnc-PURO (to shuttle from Lenti-EFS vectors into TRIPZ)
TRIPZ.CMVorf.for	CAGAGCTCGTTTAGTGAACCGTCAGATCGCACCGGTGCTGGTTTAGTGAACCGTCAGATCC	To amplify ORF-YFP (or other tags) inserts and stitch into Age1-Mlu1 digested TRIPZ
TRIPZ.marker.rev	CGGGAGGCGCCAAAACCCGGCGCGGAGGCCACGCGTGCTTTATTTGTGAAATTTGTGATGCTATTGCTTTATTTG	To amplify ORF-YFP-pA (or other tags, all have stops) inserts and stitch into Age1-Mlu1 digested TRIPZ
EFS.CMVorf.for	TTCGCAACGGGTTTGCCGCCAGAACACAGGACCGGTGCTGGTTTAGTGAACCGTCAGATCC	To amplify ORF-YFP (or other tags) inserts and stitch into Age1-BamHI digested Lenti-EFS
P2A.CMVorf.rev	TTGTTTCAGCAGAGAGAAGTTTGTTGCGCCGGATCCCTTGTACAGCTCGTCCATGCCG	To amplify ORF-YFP (only; no stop) inserts and stitch into Age1-BamHI digested Lenti-EFS

EFS.seq.for	CGTATATAAGTGCAGTAGTCGCCGTGAACGTTC	To sequence 3' of EFS promoter
CMV-F	CGCAAATGGGCGGTAGGCGTG	To sequence 3' of CMV
Blast.seq.rev	GAGATGGGGATGCTGTTGATTGTAGCCG	To sequence 5' of blasticidin-resistance cassette
Puroseq.v2.rev	CCGGGGGACGTCGTCGCGGGTGG	To sequence 5' of puromycin-resistance cassette
TurboID.Cseq.for	GCTGGAACAGGACGGAGTTATCAAACC	To sequence 3' of TurboID
GSQ.for	GGGCAAATTTCTTACGCAAGTAGAGGG	To sequence 3' of GSQ linker
TRIPZ.seqv2.for	GATGATTAATTGTCAACACGTGCTGCAGG	To sequence 3' of TRIPZ-tetO promoter
TRIPZ.seqv2.rev	CGTCTGACGTGGCAGCGCTC	To sequence 5' of TRIPZ Mlu1 site
RANGAP1.K524R.qc.for	GCTCGTGCACATGGGTCTGCTCCGCAGTGAAGACAAGGTCAAGGCCATTG	To mutate SUMOylation site in RANGAP1 (control)
RANGAP1.K524R.qc.rev	CAATGGCCTTGACCTTGTCTTCACTGCGGAGCAGACCCATGTGCACGAGC	To mutate SUMOylation site in RANGAP1 (control)
SALL1.K571R.for	CTCATACCCTTCATCCGGACGGAAGAGCCAGCC	To mutate SALL1 sumoylation consensus sites (predicted SUMOplot/GPS-SUMO/JASSA)
SALL1.K571R.rev	GGCTGGCTCTTCCGTCCGGATGAAGGGTATGAG	To mutate SALL1 sumoylation consensus sites (predicted SUMOplot/GPS-SUMO/JASSA)
SALL1.K592R.for	CCCCCAGGCTCAGTCCGGAGTGACTCCGGGGGC	To mutate SALL1 sumoylation consensus sites (predicted SUMOplot/GPS-SUMO/JASSA)
SALL1.K592R.rev	GCCCCCGGAGTCACTCCGGACTGAGCCTGGGGG	To mutate SALL1 sumoylation consensus sites (predicted SUMOplot/GPS-SUMO/JASSA)
SALL1.K982R.for	GCAGAGAAAATCATCCGGGAAGATTCTTTGGGG	To mutate SALL1 sumoylation consensus sites (predicted SUMOplot/GPS-SUMO/JASSA)
SALL1.K982R.rev	CCCCAAAGAATCTTCCCGGATGATTTTCTCTGC	To mutate SALL1 sumoylation consensus sites (predicted SUMOplot/GPS-SUMO/JASSA)
SALL1.K1086R.for	TTGTCATCTCTCATCCGGACAGAGGTCAACGGC	To mutate SALL1 sumoylation consensus sites (site from J. Kohlhase; predicted SUMOplot/GPS-SUMO/JASSA)
SALL1.K1086R.rev	GCCGTTGACCTCTGTCCGGATGAGAGATGACAA	To mutate SALL1 sumoylation consensus sites (site from J. Kohlhase; predicted SUMOplot/GPS-SUMO/JASSA)

Supplementary References:

1. Wilson, K.P., Shewchuk, L.M., Brennan, R.G., Otsuka, A.J. & Matthews, B.W. Escherichia coli biotin holoenzyme synthetase/bio repressor crystal structure delineates the biotin- and DNA-binding domains. *Proc Natl Acad Sci U S A* **89**, 9257-9261 (1992).

2. Weaver, L.H., Kwon, K., Beckett, D. & Matthews, B.W. Corepressor-induced organization and assembly of the biotin repressor: a model for allosteric activation of a transcriptional regulator. *Proc Natl Acad Sci U S A* **98**, 6045- 6050 (2001).

3. Sehnal, D., Bittrich, S., Deshpande, M., Svobodová, R., Berka, K., Bazgier, V., Velankar, S., Burley, S.K., Koča, J., Rose, A.S.. Mol* Viewer: modern web app for 3D visualization and analysis of large biomolecular structures. *Nucleic Acids Res.* **49**(W1):W431-W437 (2021).
