# Supplementary material for: Identification of proximal SUMO-dependent interactors using SUMO-ID
Source: Nat Commun. 2021 Nov 18;12:6671. doi: 10.1038/s41467-021-26807-6 (PMC8602451; doi:10.1038/s41467-021-26807-6)
Supplement: Supplementary file 3 — Description of Additional Supplementary Files [file 41467_2021_26807_MOESM3_ESM.docx]

**Description of Additional Supplementary Files**

**File Name:** Supplementary Data 1

**Description:** PML SUMO-ID LC-MS processed data.

**File Name:** Supplementary Data 2

**Description:** PML SUMO-ID GO source data.

**File Name:** Supplementary Data 3

**Description:** PML TurboID LC-MS processed data.

**File Name:** Supplementary Data 4

**Description:** PML TurboID GO source data.

**File Name:** Supplementary Data 5

**Description:** SALL1 SUMO-ID LC-MS processed data.

**File Name:** Supplementary Data 6

**Description:** SALL1 SUMO-ID GO source data.

**File Name:** Supplementary Data 7

**Description:** TP53 SUMO1-, SUMO2- and Ub-ID LC-MS

processed data.
