# Supplementary material for: Identification of proximal SUMO-dependent interactors using SUMO-ID
Source: Nat Commun. 2021 Nov 18;12:6671. doi: 10.1038/s41467-021-26807-6 (PMC8602451; doi:10.1038/s41467-021-26807-6)
Supplement: Supplementary file 11 — Reporting Summary [file 41467_2021_26807_MOESM11_ESM.pdf]

nature portfolio

Corresponding author(s): Dr. Rosa Barrio

Last updated by author(s): Sep 28, 2021

Reporting Summary

Nature Portfolio wishes to improve the reproducibility of the work that we publish. This form provides structure for consistency and transparency in reporting. For further information on Nature Portfolio policies, see our Editorial Policies and the Editorial Policy Checklist.

Statistics

For all statistical analyses, confirm that the following items are present in the figure legend, table legend, main text, or Methods section.

n/a	Confirmed	
☐	☒	The exact sample size (*n*) for each experimental group/condition, given as a discrete number and unit of measurement
☐	☒	A statement on whether measurements were taken from distinct samples or whether the same sample was measured repeatedly
☐	☒	The statistical test(s) used AND whether they are one- or two-sided<br>*Only common tests should be described solely by name; describe more complex techniques in the Methods section.*
☒	☐	A description of all covariates tested
☐	☒	A description of any assumptions or corrections, such as tests of normality and adjustment for multiple comparisons
☐	☒	A full description of the statistical parameters including central tendency (e.g. means) or other basic estimates (e.g. regression coefficient) AND variation (e.g. standard deviation) or associated estimates of uncertainty (e.g. confidence intervals)
☐	☒	For null hypothesis testing, the test statistic (e.g. *F*, *t*, *r*) with confidence intervals, effect sizes, degrees of freedom and *P* value noted<br>*Give P values as exact values whenever suitable.*
☒	☐	For Bayesian analysis, information on the choice of priors and Markov chain Monte Carlo settings
☒	☐	For hierarchical and complex designs, identification of the appropriate level for tests and full reporting of outcomes
☒	☐	Estimates of effect sizes (e.g. Cohen's *d*, Pearson's *r*), indicating how they were calculated

Our web collection on statistics for biologists contains articles on many of the points above.

Software and code

Policy information about availability of computer code

Data collection	timsTOF Pro with PASEF, Bruker Daltonics<br>Exploris 480 mass spectrometer, ThermoFisher<br>Leica SP8 Lightning confocal mycroscope, 63x Plan ApoChromat NA1.4
Data analysis	MaxQuant v1.6.14.0 and v1.6.17.0, Andromeda (RRID: SCR_014485)<br>Perseus v1.6.14.0 (RRID: SCR_015753)<br>ImageJ v2.0.0-rc-69/1.52n (RRID: SCR_003070)<br>JACoP v2.1.1 [https://imagejdocu.tudor.lu/doku.php?id=plugin:analysis:jacop_2.0:just_another_colocalization_plugin:start]<br>Colocalization_Colormap v12_11_2019 [https://sites.google.com/site/colocalizationcolormap/home]<br>Coloc 2 v3.0.5<br>GraphPad Prism 8 v.8.4.3 (RRID: SCR_015807)<br>g:Profiler web server version e104_eg51_p15_3922dba [https://biit.cs.ut.ee/gprofiler/gost]<br>Cytoscape v3.7.2 (RRID: SCR_003032)<br>STRING v1.4.2 (RRID: SCR_005223)<br>MCODE v1.5.1 (RRID: SCR_015828)<br>Python v2.7.5 (RRID:SCR_008394)<br>R v.3.6.0 (RRID:SCR_001905)<br>SIM enrichment script (Source Data file)

For manuscripts utilizing custom algorithms or software that are central to the research but not yet described in published literature, software must be made available to editors and reviewers. We strongly encourage code deposition in a community repository (e.g. GitHub). See the Nature Portfolio guidelines for submitting code & software for further information.

Data

Policy information about availability of data

All manuscripts must include a data availability statement. This statement should provide the following information, where applicable:

- Accession codes, unique identifiers, or web links for publicly available datasets
- A description of any restrictions on data availability
- For clinical datasets or third party data, please ensure that the statement adheres to our policy

All data supporting the findings are provided within the paper, the Supplementary Data and the Supplementary Information. The fasta file of the human proteome (Uniprot filtered reviewed H. sapiens proteome, UP000005640) was downloaded from Uniprot [https://www.uniprot.org/uniprot/?query=proteome:UP000005640%20reviewed:yes]. In addition, the mass spectrometry proteomics raw data corresponding to PML SUMO-ID, PML-TurboID, SALL1 SUMO-ID and TP53 SUMO-ID/Ub-ID experiments have been deposited to the ProteomeXchange Consortium via the PRIDE partner repository with the dataset identifiers PXD021770 [https://www.ebi.ac.uk/pride/archive/projects/PXD021770], PXD021809 [https://www.ebi.ac.uk/pride/archive/projects/PXD021809], PXD021923 [https://www.ebi.ac.uk/pride/archive/projects/PXD021923] and PXD027759 [https://www.ebi.ac.uk/pride/archive/projects/PXD027759], respectively. Processed LC-MS/MS data as well as their corresponding gene ontology source data are provided as Supplementary Data files. Source data are provided with this manuscript

Field-specific reporting

Please select the one below that is the best fit for your research. If you are not sure, read the appropriate sections before making your selection.

- [x] Life sciences
- [ ] Behavioural & social sciences
- [ ] Ecological, evolutionary & environmental sciences

For a reference copy of the document with all sections, see nature.com/documents/nr-reporting-summary-flat.pdf

Life sciences study design

All studies must disclose on these points even when the disclosure is negative.

Sample size	Sample size for LC-MS/MS and corresponding Western Blots was set up to 80.000.000 cells (4*15cm dishes) per replicate. This was determined by measuring the elution efficiency of pull-down experiments (comparing different sample sizes) and by identifying IDs with pilot LC-MS/MS experiments. The sample size was sufficient and efficient in terms of amount of beads/elution volume ratio, as elution volume restricts the sample size that can be loaded into the gel and analyzed by LC-MS/MS. For cell imaging, at least 4 independent acquisitions per coverslip were taken. For the SIM enrichment analysis, the amount of generated lists was settled to 1000, which was sufficient to confirm the Gaussian distribution of data (d'Agostino and Pearson normality test, p-value = 0.15). Other information on sample sizes for each experiment is described within the figure legends and Methods section.
Data exclusions	For LC-MS/MS data analysis, the exclusion criteria were pre-established and are commonly used for improving the confidence of identifications: proteins detected with at least 2 peptides (except when otherwise specified) and in at least 2 of the 3 replicates were included. Contaminants, only identified by site and reverse identified proteins were also excluded. During the SIM enrichment analysis, three outliers (lists 46, 782, 794; ROUT method, Q=1%) were excluded. For PML bodies comparisons, outliers were also removed (ROUT method, Q=1%).
Replication	For all LC-MS/MS experiments, three independent replicates per condition were analyzed, and all attempts at replication were successful. For the rest, the number of times that each experiment was performed is described in the associated figure legends. All attempts at replication were successful.
Randomization	For the SIM enrichment analyses, 1000 lists of the same size as PML SUMO-ID list were generated randomly. The script is available in the source data file. For cell imaging experiments, at least 4 pictures per cover slip were randomly taken. For the rest of the experiments, randomization is not applicable as no sub-sampling is performed: all lysates are analyzed by WB and replicates were pulled for validation experiments.
Blinding	LC-MS/MS samples were encoded and blindly acquired and analyzed in two different facilities. Investigators were blinded to group allocation during data collection. For the rest of the experiments, the investigators were not blinded to group allocations. Although samples were always encoded, blinding was not possible for terms of logistics: logical and visual order of samples in a gel for Western Blotting, order of replicates in microscope slices, etc... Blinding in those experiment was not relevant and proper laboratory practices were employed: samples of the same experiment were collected in the same conditions, replicates were performed and proper positive and negative controls were always included.

Reporting for specific materials, systems and methods

We require information from authors about some types of materials, experimental systems and methods used in many studies. Here, indicate whether each material, system or method listed is relevant to your study. If you are not sure if a list item applies to your research, read the appropriate section before selecting a response.

Materials & experimental systems

n/a	Involved in the study
[ ]	[x] Antibodies
[ ]	[x] Eukaryotic cell lines
[x]	[ ] Palaeontology and archaeology
[x]	[ ] Animals and other organisms
[x]	[ ] Human research participants
[x]	[ ] Clinical data
[x]	[ ] Dual use research of concern

Methods

n/a	Involved in the study
[x]	[ ] ChIP-seq
[x]	[ ] Flow cytometry
[x]	[ ] MRI-based neuroimaging

Antibodies

Antibodies used

Goat polyclonal anti-biotin HRP-linked (Cell Signaling; Cat#7075S; RRID: AB_10696897), Mouse monoclonal anti-Myc-Tag clone 9B11 (Cell Signaling; Cat#2276S; RRID: AB_331783), Mouse monoclonal anti-Flag-Tag clone M2 (Sigma-Aldrich; Cat#F1804; RRID: AB_262044), Rabbit polyclonal anti-BirA (SinoBiological; Cat#11582-T16), Rabbit polyclonal anti-GFP (Santa Cruz; Cat#sc-8334; RRID:AB_641123), Mouse monoclonal anti-B23 clone E-3 (Santa Cruz; Cat#sc-271737; RRID:AB_10708848), Mouse monoclonal anti-SC35 clone αSC35 (BD Biosciences; Cat#556363; RRID:AB_396388), Rabbit polyclonal anti-PML (Bethyl; Cat#A301-167A; RRID:AB_873108), Rabbit polyclonal anti-GTF2I (Sigma-Aldrich; Cat#HPA026638; RRID: AB_1857959), Mouse monoclonal anti-SUMO2 clone 8A2 (DSHB; Cat#SUMO-2 8A2; RRID:AB_2198421), Rabbit polyclonal anti-PML (Proteintech; Cat#21041-1-AP; RRID:AB_2878799), Rabbit polyclonal anti-IRF2BP2 (Proteintech; Cat#18847-1-AP; RRID: AB_10598163), Rabbit polyclonal anti-UBC9 (Proteintech; Cat#14837-1-AP; RRID: AB_2272479), Rabbit polyclonal anti-TRIM24 (Proteintech; Cat#14208-1-AP; RRID: AB_2256646), Rabbit polyclonal anti-TRIM33 (Proteintech; Cat#55374-1-AP; RRID: AB_11183035), Rabbit polyclonal anti-PIAS2 (Proteintech; Cat#16074-1-AP; RRID: AB_2164208), Rabbit polyclonal anti-PIAS4 (Proteintech; Cat#14242-1-AP; RRID: AB_2164365), Rabbit polyclonal anti-GATAD2B (Proteintech; Cat#25679-1-AP), Rabbit polyclonal anti-MTA2 (Proteintech Cat#17554-1-AP; RRID: AB_10638601), Rabbit polyclonal anti-RBBP4 (Proteintech; Cat#20364-1-AP; RRID: AB_10694151), Rabbit polyclonal anti-CBX4 (Proteintech; Cat#18544-1-AP; RRID:AB_2878552), Mouse monoclonal anti-GAPDH clone 1E6D9 (Proteintech; Cat#60004-1-Ig; RRID: AB_2107436), Mouse monoclonal anti-beta-Actin clone 2D4H5 (Proteintech; Cat#66009-1-Ig; RRID: AB_2687938), Mouse monoclonal anti-alpha-Tubulin clone 1E4C11 (Proteintech; Cat#66031-1-Ig; RRID: AB_11042766), Rabbit monoclonal anti-alpha-Actinin clone D6F6 (Cell Signaling Cat#6487S; RRID: AB_11179206), Goat polyclonal anti-Mouse HRP-linked (Jackson ImmunoResearch; Cat#115-035-062; RRID: AB_2338504), Goat polyclonal anti-Rabbit HRP-linked (Jackson ImmunoResearch; Cat#111-035-045; RRID: AB_2337938), Goat polyclonal anti-Rabbit Alexa Fluor 488-linked (Invitrogen; Cat#A-11034; RRID: AB_2576217), Goat polyclonal anti-Mouse Alexa Fluor 568-linked (Invitrogen; Cat#A-11031; RRID: AB_144696), Goat polyclonal anti-Rabbit Alexa Fluor 568-linked (Invitrogen; Cat#A-11036; RRID: AB_10563566), Streptavidin Alexa Fluor 594-linked (Jackson ImmunoResearch; Cat#016-290-084; RRID: AB_2337247).

Validation

Goat polyclonal anti-biotin HRP-linked (Cell Signaling; Cat#7075S;), Mouse monoclonal anti-Myc-Tag clone 9B11 (Cell Signaling; Cat#2276S) and Rabbit polyclonal anti-BirA (SinoBiological; Cat#11582-T16) were validated in this study using overexpression and knock-in approaches, and used for Western Blot (WB) and Immunofluorescence (IF) experiments. We also validated that Rabbit polyclonal anti-BirA (SinoBiological; Cat#11582-T16) antibody recognizes both N194TurboID and C195TurboID fragments by WB and IF. Mouse monoclonal anti-Flag-Tag clone M2 (Sigma-Aldrich; Cat#F1804), Rabbit polyclonal anti-GFP (Santa Cruz; Cat#sc-8334), Rabbit polyclonal anti-PML (Bethyl; Cat#A301-167A) and Rabbit polyclonal anti-PML (Proteintech; Cat#21041-1-AP) were validated in this study using overexpression and knock-in approaches, and used for WB. Mouse monoclonal anti-B23 clone E-3 (Santa Cruz; Cat#sc-271737): several applications validated and published on the website [https://www.scbt.com/p/b23-antibody-e-3], including WB, Immunohistochemistry (IHC) and IF. Used for IF in this study. Mouse monoclonal anti-SC35 clone αSC35(BD Biosciences; Cat#556363): several published validations for WB, IF and Immunoprecipitation (IP) on the website [https://www.bdbiosciences.com/en-us/products/reagents/microscopy-imaging-reagents/immunofluorescence-reagents/purified-mouse-anti-sc35.556363]. Used for IF in this study. Mouse monoclonal anti-SUMO2 clone 8A2 (DSHB; Cat#SUMO-2 8A2): several applications validated and published on the website [https://dshb.biology.uiowa.edu/SUMO-2-8A2] including WB and IF. Used for IF in this study. Rabbit polyclonal anti-GTF2I (Sigma-Aldrich; Cat#HPA026638): several applications validated and published on The Human Protein Atlas [https://www.proteinatlas.org/ENSG00000263001-GTF2I/antibody], including IF and WB. Used for IF and WB in this study. Rabbit polyclonal anti-IRF2BP2 (Proteintech; Cat#18847-1-AP): several applications validated and published on the website [https://www.ptglab.com/products/IRF2BP2-Antibody-18847-1-AP.htm#publications], including IF and WB. Used for WB and IF in this study. Rabbit polyclonal anti-UBC9 (Proteintech; Cat#14837-1-AP): several applications validated and published on the website [https://www.ptglab.com/products/UBE2I-Specific-Antibody-14837-1-AP.htm] including WB and IHC. Used for WB and IF in this study. Rabbit polyclonal anti-TRIM24 (Proteintech; Cat#14208-1-AP): several applications validated and published on the website [https://www.ptglab.com/products/TRIM24-Antibody-14208-1-AP.htm] including WB, IHC and IF. Used for WB and IF in this study. Rabbit polyclonal anti-TRIM33 (Proteintech; Cat#55374-1-AP): validated and published on the website [https://www.ptglab.com/Products/TRIM33-Antibody-55374-1-AP.htm]. Used for WB and IF in this study. Rabbit polyclonal anti-PIAS2 (Proteintech; Cat#16074-1-AP): validated and published on the website [https://www.ptglab.com/products/PIAS2-Antibody-16074-1-AP.htm] for WB, IF and ELISA. Used for WB and IF in this study. Rabbit polyclonal anti-PIAS4 (Proteintech; Cat#14242-1-AP): validated and published on the website [https://www.ptglab.com/products/PIAS4-Antibody-14242-1-AP.htm] for WB, IF and ELISA. Used for WB and IF in this study. Rabbit polyclonal anti-GATAD2B (Proteintech; Cat#25679-1-AP): validated and published on the website [https://www.ptglab.com/products/GATAD2B-Antibody-25679-1-AP.htm] for WB, IF and ELISA. Used for WB in this study. Rabbit polyclonal anti-MTA2 (Proteintech Cat#17554-1-AP): several applications validated and published on the website [https://www.ptglab.com/products/MTA2-Antibody-17554-1-AP.htm] including WB. Used for WB in this study. Rabbit polyclonal anti-RBBP4 (Proteintech; Cat#20364-1-AP): several applications validated and published on the website [https://www.ptglab.com/products/RBBP4-Antibody-20364-1-AP.htm] including WB. Used for WB in this study. Rabbit polyclonal anti-CBX4 (Proteintech; Cat#18544-1-AP): validated and published in the website [https://www.ptglab.com/products/CBX4-Antibody-18544-1-AP.htm] for WB, IF and ELISA. Used for IF in this study. Mouse monoclonal anti-GAPDH clone 1E6D9 (Proteintech; Cat#60004-1-Ig): several applications validated and published in the website [https://www.ptglab.com/products/GAPDH-Antibody-60004-1-Ig.htm] including WB. Used for WB in this study. Mouse monoclonal anti-beta-

Actin clone 2D4H5 (Proteintech; Cat#66009-1-Ig): several applications validated and published in the website [https://www.ptglab.com/products/Pan-Actin-Antibody-66009-1-Ig.htm] including WB. Used for WB in this study. Mouse monoclonal anti-alpha-Tubulin clone 1E4C11 (Proteintech; Cat#66031-1-Ig): several applications validated and published in the website [https://www.ptglab.com/products/tubulin-Alpha-Antibody-66031-1-Ig.htm] including WB. Used for WB in this study. Rabbit monoclonal anti-alpha-Actinin clone D6F6 (Cell Signaling Cat#6487S): validated and published in the website [https://www.cellsignal.com/products/primary-antibodies/a-actinin-d6f6-xp-rabbit-mab/6487] for WB and IF. Used in this study for WB.

Eukaryotic cell lines

Policy information about cell lines

Cell line source(s)	U2OS (ATCC; HTB-96; RRID: CVCL_0042)<br>HEK 293FT (Invitrogen; R70007; RRID: CVCL_6911)<br>hTERT-RPE1 (ATCC; CRL-4000; RRID: CVCL_4388)
Authentication	U2OS, HEK 293FT and hTERT-RPE1 cells were used at low passage after purchase or acquisition.<br>CRISPR/Cas9 cell lines were verified by sequencing.<br>All generated stable cell lines were regularly verified by WB.
Mycoplasma contamination	All the cell lines used in the study were negative for mycoplasma contamination
Commonly misidentified lines<br>(See ICLAC register)	No cell lines used are listed in the database of commonly misidentified cell lines.
