# Supplementary material for: Identification of proximal SUMO-dependent interactors using SUMO-ID
Source: Nat Commun. 2021 Nov 18;12:6671. doi: 10.1038/s41467-021-26807-6 (PMC8602451; doi:10.1038/s41467-021-26807-6)

Figure 2b

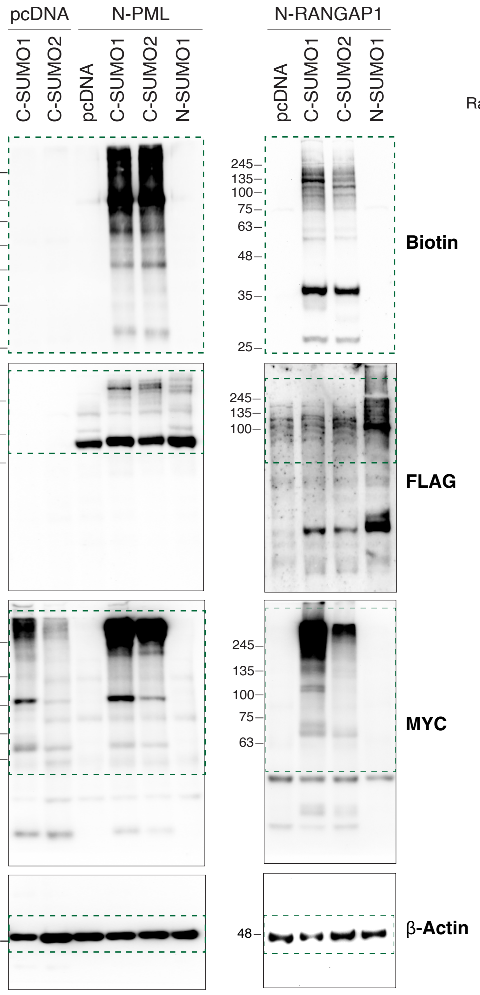

Figure 2d

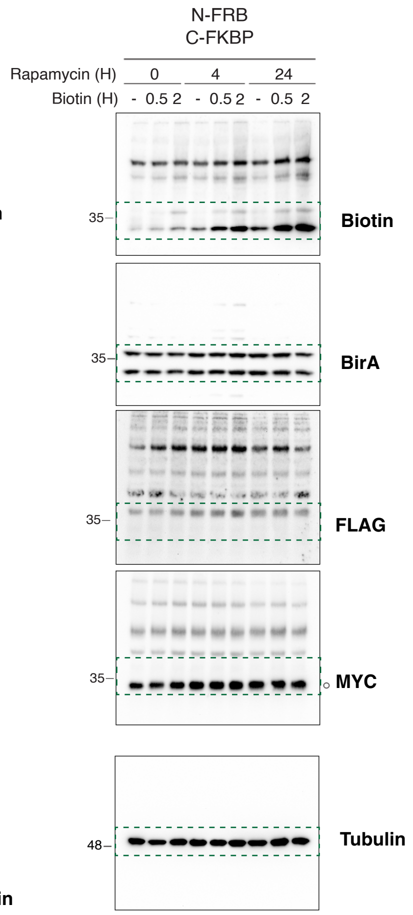

Figure 3a

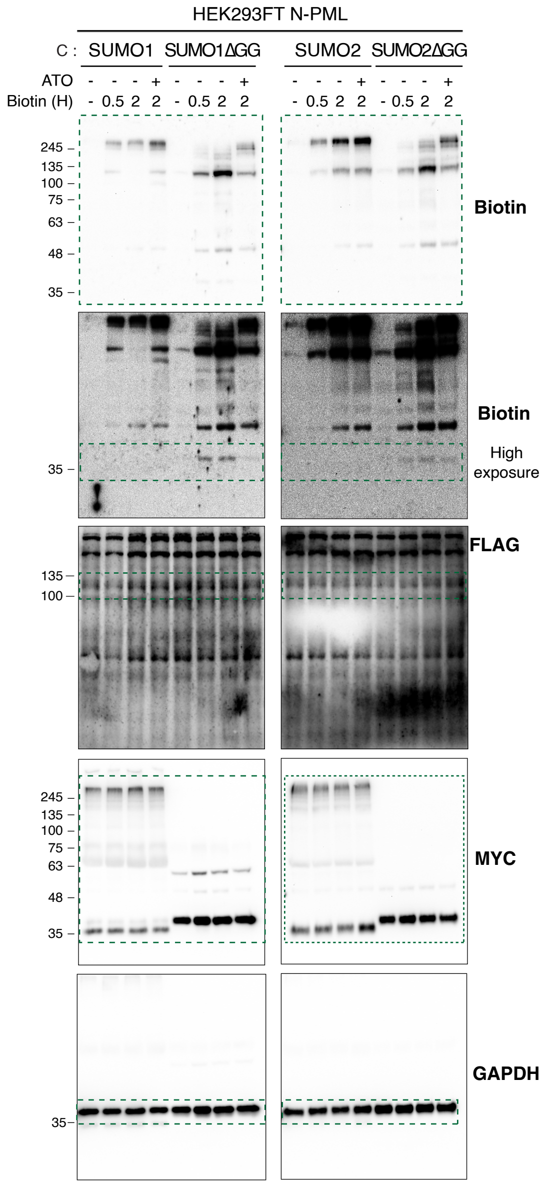

Figure 4b

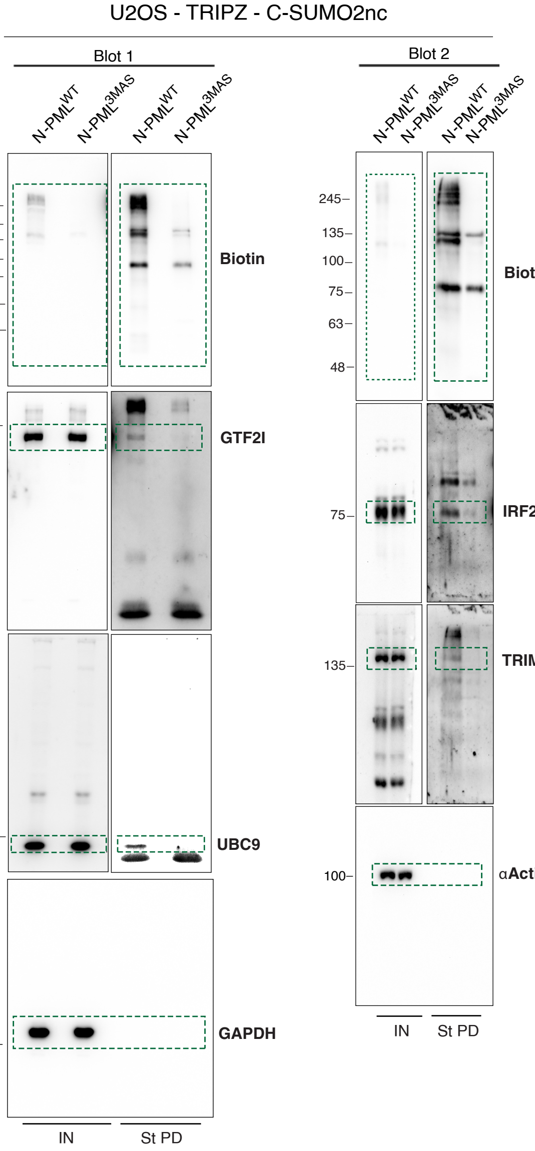

Figure 3b

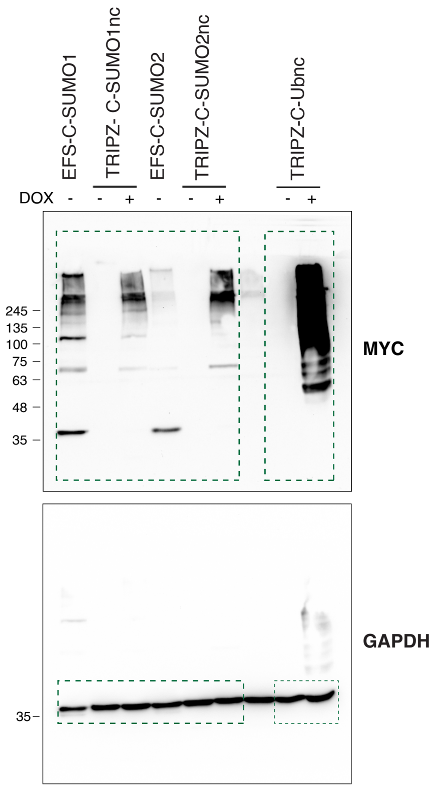

Figure 3c

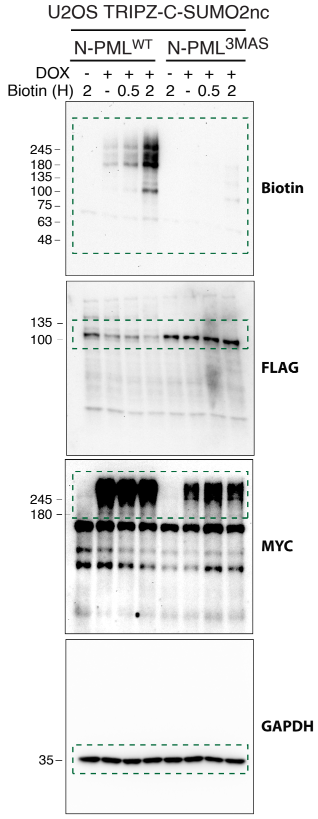

Figure 7b

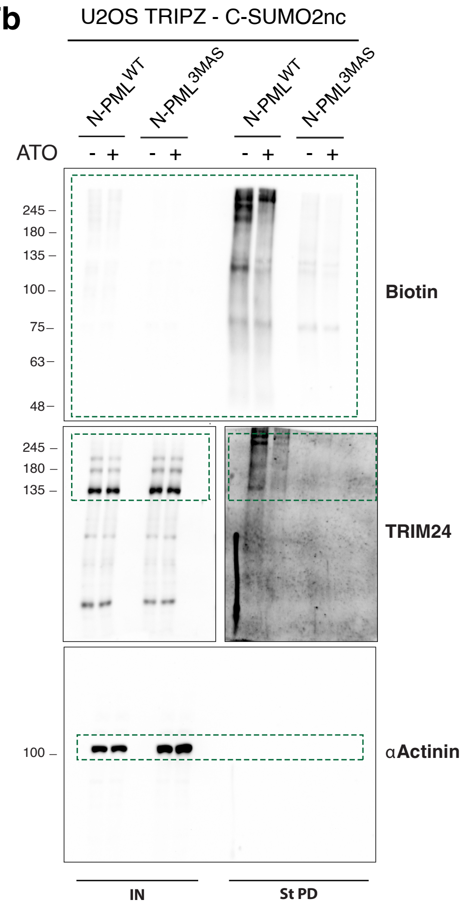

Figure 8a

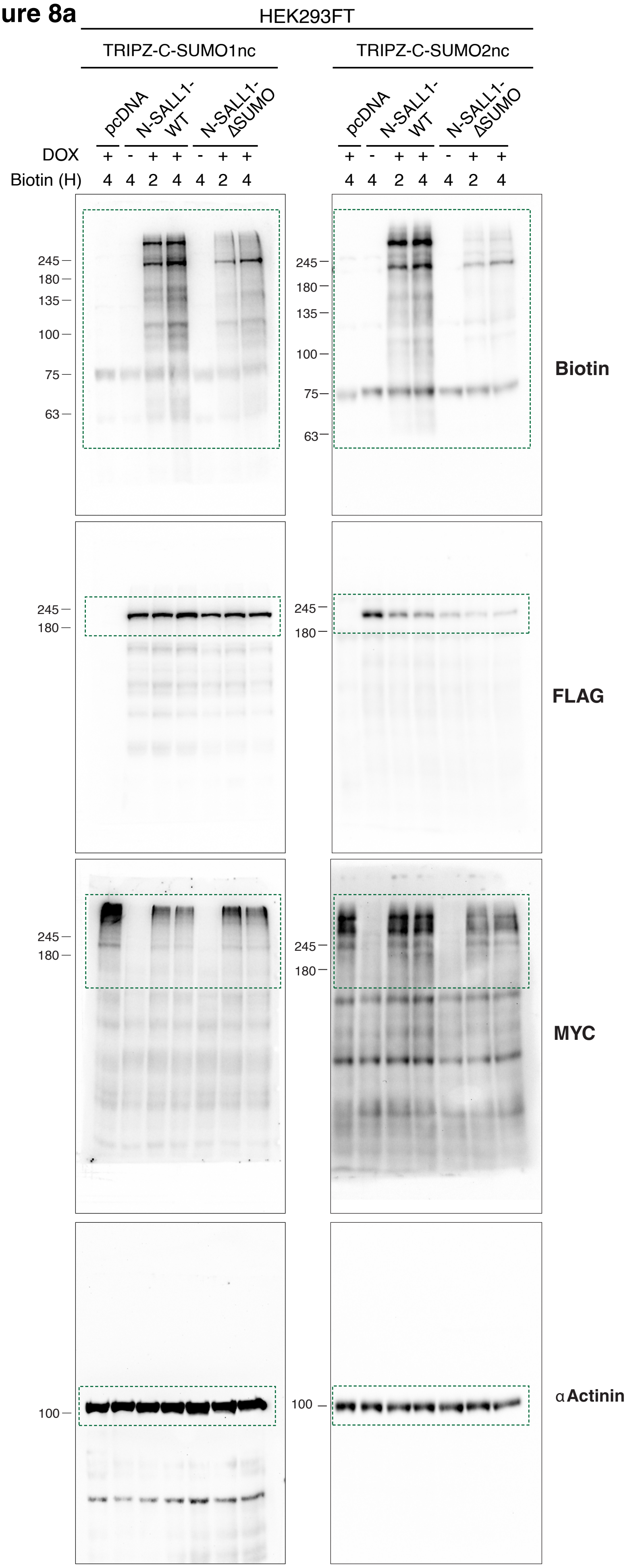

Figure 8d

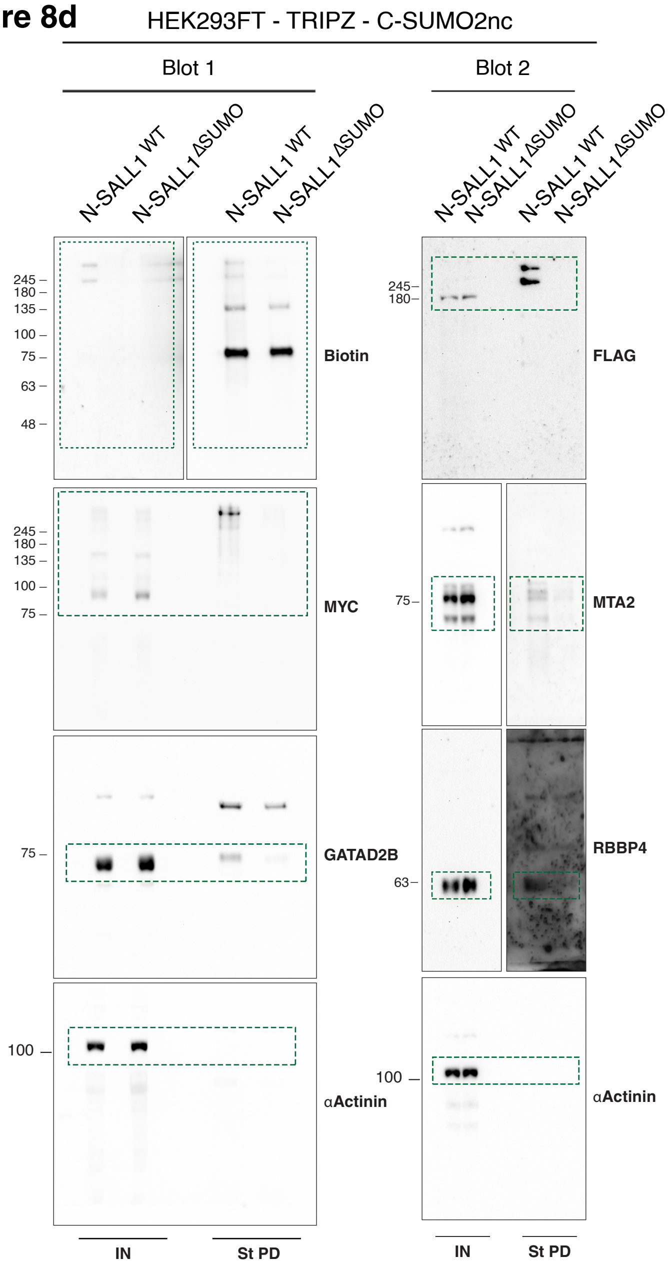

Figure 9a

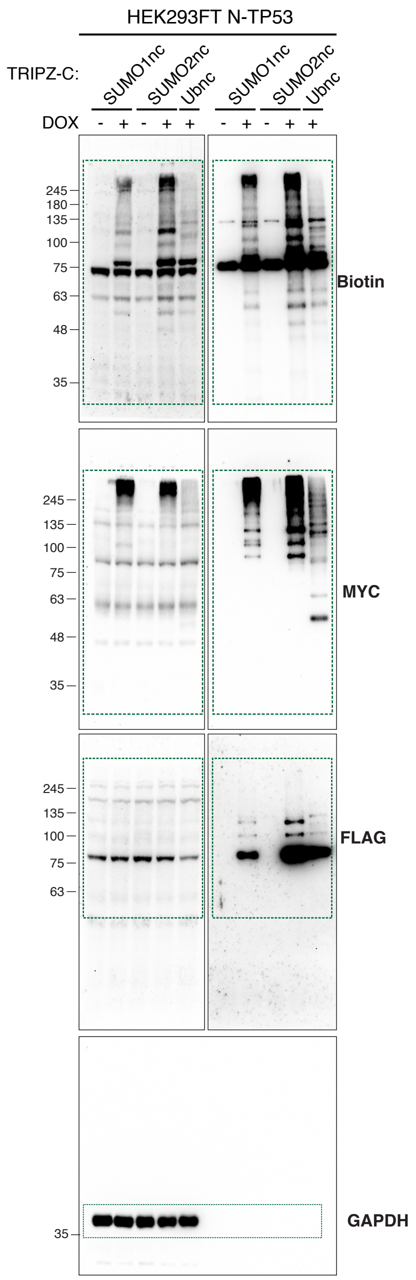

Supplementary Figure 1b

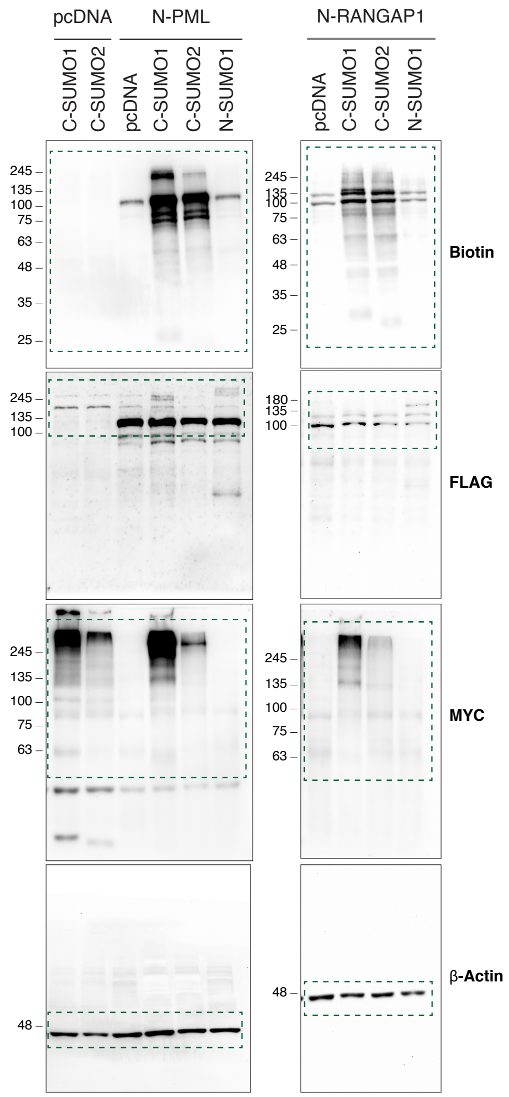

Supplementary Figure 2

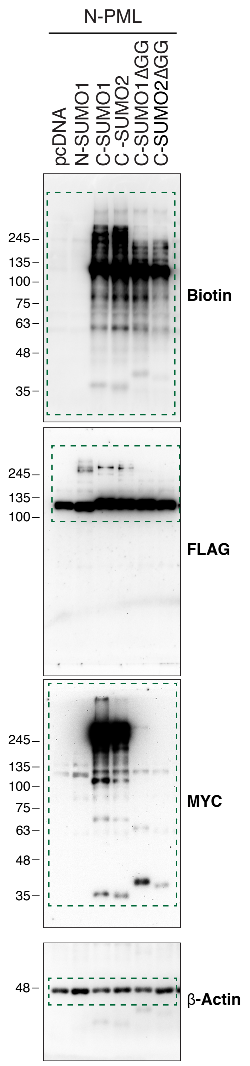

Supplementary Figure 3a

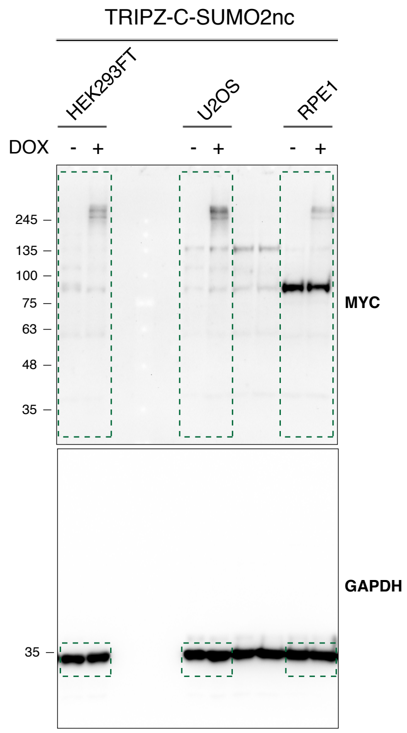

Supplementary Figure 5

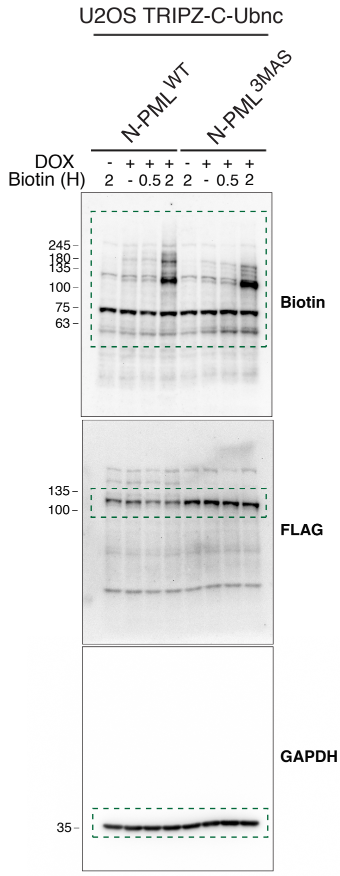

Supplementary Figure 6a

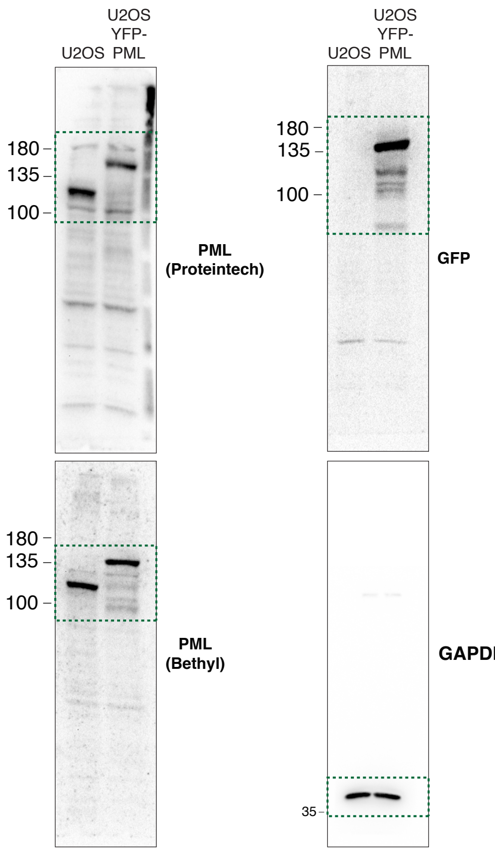

Supplement: Supplementary file 12 — Source data [file 41467_2021_26807_MOESM12_ESM.zip › Source_data_file/Uncropped_blots/Uncropped_blots.pdf]
