# Supplementary figures and images for: Identification of proximal SUMO-dependent interactors using SUMO-ID
Source: Nat Commun. 2021 Nov 18;12:6671. doi: 10.1038/s41467-021-26807-6 (PMC8602451; doi:10.1038/s41467-021-26807-6)

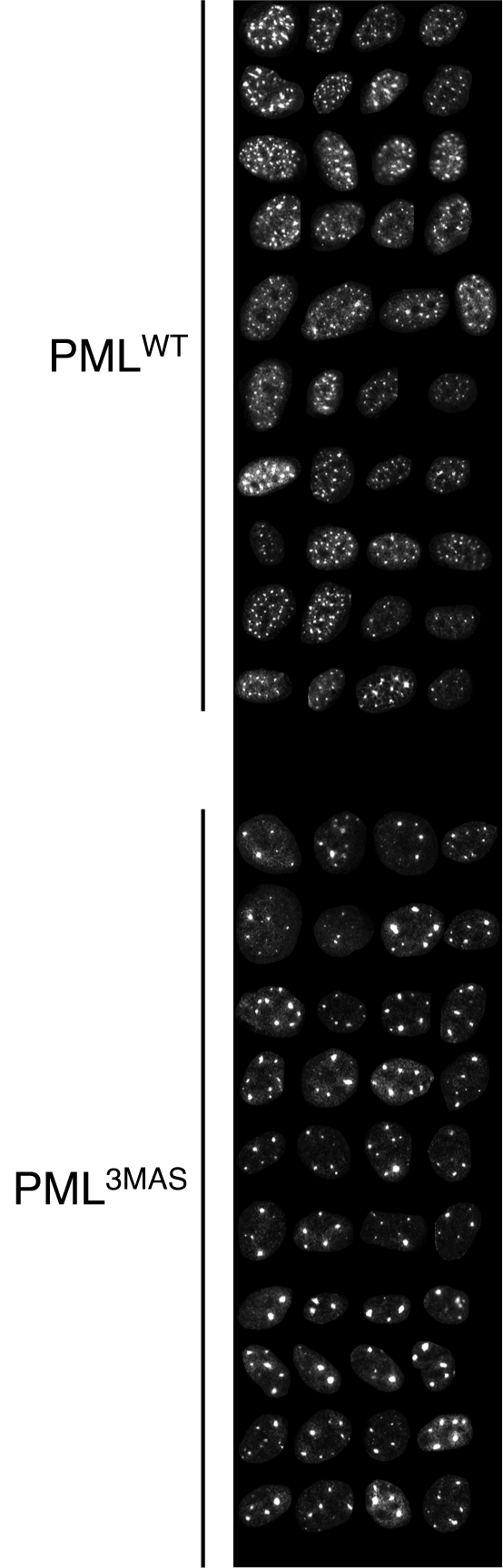

Supplement: Supplementary file 12 — Source data [file 41467_2021_26807_MOESM12_ESM.zip › Source_data_file/Supplementary_Fig_7b/Supplementary_Fig_7b_source_cells.tif]
